# Getting to grips with the process of decision-making in long-term care. Descriptive cases illustrate the chaotic reality of the construction of preferences

**DOI:** 10.1371/journal.pone.0217338

**Published:** 2019-05-24

**Authors:** Catharina M. van Leersum, Albine Moser, Ben van Steenkiste, Judith R. L. M. Wolf, Trudy van der Weijden

**Affiliations:** 1 Department of Family Medicine, CAPHRI School for Public Health and Primary Care, Maastricht University Medical Centre, Maastricht, The Netherlands; 2 Research Centre for Autonomy and Participation of Persons with a Chronic Illness, Zuyd University of Applied Sciences, Heerlen, The Netherlands; 3 Impuls–Netherlands Center for Social Care Research, Radboud Institute for Health Sciences, Radboud University Medical Center, Nijmegen, The Netherlands; University of Malaya, MALAYSIA

## Abstract

**Background:**

Clients facing decision-making for long-term care are in need of support and accessible information. Construction of preferences, including context and calculations, for clients in long-term care is challenging because of the variability in supply and demand. This study considers clients in four different sectors of long-term care: the nursing and care of the elderly, mental health care, care of people with disabilities, and social care. The aim is to understand the construction of preferences in real-life situations.

**Method:**

Client choices were investigated by qualitative descriptive research. Data were collected from 16 in-depth interviews and 79 client records. Interviews were conducted with clients and relatives or informal caregivers from different care sectors. The original client records were explored, containing texts, letters, and comments of clients and caregivers. All data were analyzed using thematic analysis.

**Results:**

Four cases showed how preferences were constructed during the decision-making process. Clients discussed a wide range of challenging aspects that have an impact on the construction of preferences, e.g. previous experiences, current treatment or family situation. This study describes two main characteristics of the construction of preferences: context and calculation.

**Conclusion:**

Clients face diverse challenges during the decision-making process on long-term care and their construction of preferences is variable. A well-designed tool to support the elicitation of preferences seems beneficial.

## Introduction

This study considers clients in four sectors of long-term care: nursing and care of the elderly, mental health care, care of people with disabilities, and social care. These clients receive long-term care, which is care that is provided for at least six months [1] for reasons of ageing, disability, chronic illness, or in any situation that limits their ability to care for themselves and manage activities, e.g. washing, grocery shopping or work. Care can be provided to these clients in any setting, ranging from home care, to care facilities or nursing home care [2]. In making the decision to stay at home or move to a care facility, there are a range of care and social services for the clients who need assistance. How clients get to grips with the decision-making process concerning long-term care, the barriers they might face, and the assistance they need is currently unknown.

Long-term care should facilitate adjustment of functioning and maintaining quality of life instead of returning to a ‘normal’ functioning level [1]. A person’s life or lifestyle might change, instead of being self-reliant or asking family for help, the client may become dependent on caregivers. Care often takes place in the personal environment, making it important to build a relationship of trust with the caregiver. This requires information about care other than quality indicators of care organizations provided by the Dutch government. In the Netherlands, in order to improve personalized care, discussions based on dialogue between the local authority or a care organization and the client have been introduced. Clients get the opportunity to explain their needs and wishes for assistance and care, and discuss the possibility of relatives being able to provide assistance [3]. Discussing preferences and possible solutions for the fulfilment of needs may also stimulate a client’s self-reliance [4, 5].

Decisions concerning the long-term usually have a powerful impact on someone’s quality of daily life, as the outcome is unpredictable and the cure might be care and support [5, 6]. Clients incorporate all kinds of factors that influence their well-being into their decision, including health needs, living situation, and daily activities. If preferences are incorporated into the decision-making process, a patient-centred type of care could be provided to help clients adapt to the care and its influence on their lifestyle [5].

When defining preferences, intuition plays an important role. However, it is possible that intuition might introduce misconceptions into the decision-making process [7]. Although situation has a strong influence on intuition, other factors such as information received before making the choice and hearing the experiences of others, can have an impact on decision-making [8]. Taking the time to make a decision, gives clients the opportunity to construct preferences in order to overcome possible misconceptions induced by intuition [7]. However, the dynamic property of preferences means that the time at which a certain medical/psycho-social situation induces a change in preferences is unknown [9].

Clients implicitly or explicitly construct preferences during the decision-making process. Although clients receive information about standardized quality aspects, these official sources of information do not influence the decision-making process [10]. Clients prefer information from informal sources and experiences of others. In order to understand the construction of preferences, research into the decision-making process, the information clients actually need, and the support they prefer to receive during the decision-making process is needed [10].

The concept of preference construction enables a deeper understanding of the decision-making process [11]. Warren et al. regard context and calculation as two important aspects of the construction of preferences [12]. The context, which happens unconsciously, is the intuition created by a specific situation. The context is the environment in which a person imparts a meaning to a decision. Preferences are context-sensitive and therefore continuously being reconstructed when the context changes. Preferences differ depending on the context. Calculation refers to the slower construction of preferences, and refers to the integration of knowledge to form a preference while making a decision. It is a conscious process, which depends on reflection on aspects such as background and experiences, knowledge acquisition (information sources), rational arguments, and rules and regulations. Although the context always influences the construction of preferences, calculation occurs less frequently, because it requires more time [12]. When time is available, calculation can have an impact, because it enables clients to reflect on their intuition and consider options.

The aim of this paper is to understand the construction of preferences in real-life situations during the decision-making process of clients in need of long-term care and answers the question *“*What are the factors that influence the construction of preferences for clients during the decision-making process on long-term care?” This study does not provide a retrospective view, but a view on the challenges clients experience during the decision-making process.

## Method

### Design

This study has a qualitative descriptive research design. The methodological orientation underpinning the approach was a naturalistic inquiry [13] to explore the multiple and subjective experiences, preferences, and events of clients in long-term care during the decision-making process. To explore the construction of preferences during decision-making regarding long-term care, 16 in-depth interviews and 79 client records were analyzed and distributed over four care sectors: the nursing and care of elderly, mental health care, care of people with disabilities, and social care.

### Ethical approval

Ethical approval was obtained from the Zuyderland Zuyd Ethics Committee (dossier-number 2015–1791).

### Interviews

#### Participants

Participants were recruited by formal caregivers from 12 care organizations that provide long-term care in four provinces of the Netherlands. The participants were selected trough convenience sampling. Caregivers identified 16 participants from the database of their organization. Participants were either actively involved in the decision-making process or had recently made a decision in a non-acute situation (less than six weeks previously). Clients with acute psychiatric symptoms were excluded. Eight interviews were conducted with family relatives, when they considered an in-depth interview to be too burdensome for the client.

All participants received a letter from their professional caregiver. The letter included information about the research, its confidential character, and the informed consent form. The caregivers forwarded the personal data to the researchers. Then, the researcher conducting the interview contacted the participants to explain the project and the interview procedure in more detail. In a second call, a few days later, an appointment was arranged. The participants were informed they could withdraw from the study at any point, gave written informed consent, and the data were anonymized and data confidentiality was maintained.

#### Data collection

Data were collected between September 2015 and June 2016. Four experienced researchers conducted the in-depth semi-structured interviews. They evaluated the process and results regularly in order to align the data collection process. The researchers had weekly meetings, summarised in minutes. At the start of the study, the recruitment, procedures, and information letters were discussed in the team. Subsequently, the interview guide was aligned within the team and the interview schedule was continuously refined based on reflection and discussion of the data as it was being collected. During data analysis, the findings were exchanged and discussed in the team. All participants were interviewed one time at the client’s home to ensure an environment that was comfortable to participants. The interviews lasted between 30 and 60 minutes.

The interview guide consisted of open-ended questions developed by the researchers oriented to themes from the literature as described in the introduction (interview guide in S1 File). Seven clients from the client panels of care organizations reviewed the interview guide. In the early version, they found that the language was too difficult and questions addressing emotional consequences during the decision-making were missing. The guide was adjusted in accordance with their feedback. The questions focused on:

1. The current situation and phase of decision-making of the participant.
2. The events, contacts, and aspects crucial for the decision a client was making.
3. The client’s need for support during the decision-making process.
4. The fit of the actual choice with the client’s preferences.
5. The information sources the client used and the relevance of these sources.

Field notes were taken during the interviews and audiotapes were transcribed verbatim by members of the research team.

### Client records

#### Participants

Seventy-nine client records were selected by formal caregivers of 12 care organizations in long-term care. The inclusion criteria for these records were similar to those used for inclusion of clients for the interviews. Additionally, the last update to the records had to have been made less than six months previously. Caregivers were asked to select ten records of clients meeting the inclusion criteria. These clients were not included in the interviews.

#### Data collection

Between January and March 2016, client records were selected from the current databases of the organizations. The records included organizational files, a care plan, life plan or activity plan and sometimes contained notes on deliberations or arguments on decisions. The content of the records was comparable between organizations and were therefore a valid and rich source of information about construction of preferences. The care organizations anonymized all files in preparation of analysis. The same four researchers who conducted the interviews reviewed the client records.

### Data analysis

The analysis consisted of the same two steps for the interviews and the client records. First, inductive coding to observe and combine different aspects into overarching themes. Second, deductive coding based on the theory of construction of preferences, using context and calculation. The first step in the inductive coding was open coding to identify all themes [14]. The main themes within the coding three appeared to be context, life story, and care needs.

The transcripts were read and codes were accorded to the client quotes. Two researchers coded the files of six interviews and client records and then compared the codes. Then all codes emerging from the transcripts were combined into a list of overarching themes to reduce the number of codes [14]. One researcher coded all other files and discussed the findings with the research team at weekly meetings. The researchers used software package NVivo 11. Data saturation was reached when no new themes emerged. The analysis resulted in four narratives of four personas.

### Trustworthiness

To establish credibility [13], two methods of data collection were combined: client records and in-depth interviews (method triangulation). Four researchers collected and analyzed the data. The data collection process was discussed at regular meetings. The data were read and analyzed in several steps to compare and discuss contrasting findings (investigator triangulation). The findings were discussed with all participants during an invitational conference (member check).

The thick description included the research settings, sampling method, participant selection, the inclusion and exclusion criteria, data collection procedure, interviewing methods, and the questions of the interview guide. This thick description was made to ensure transparency, validity, and transferability to other settings [13].

## Results

### Participants

Sixteen participants were included in the interviews, 57% male and 43% female. The participants were between 20 and 93 years old. One third of them lived alone and the others lived with a partner or parents. Forty percent had never received education or had a low educational level. The interviews included seven clients in the nursing and care of the elderly sector, four clients in the care of people with disabilities sector, one client in the mental health care sector, one client in the social care sector, and three clients received help from more than one care sector. Three interviews were held with the client alone, eight interviews with the client’s relatives, and five interviews with both the client and relative.

The 79 client records included 46% male and 54% female, and 47% lived in a specialized care facility and 53% lived independently. There were 23 clients in the nursing and care of the elderly sector, 20 clients in the care of people with disabilities sector, 10 clients in the mental health care sector, and 26 clients in the social care sector.

### Reflections on the cases

Four case based on real-life experiences were used to illustrate the construction of preferences. These cases are representative for the whole data set and illustrate how clients try to get to grips with the decision-making process, the barriers they encountered, and areas where assistance might be needed. The case descriptions are given in Boxes 1–4. Each box is followed by a reflection on the cases based on the analysis using the concept of preference construction. The influence of context or calculation on each case is determined and described.

**Box 1. Case description Annie (nursing care and care of the elderly)** Annie, an 83-year-old woman who initially lived independently in her own home with some support from her children, had been searching for a care facility since being diagnosed with obstructive sleep apnoea syndrome (OSAS). Annie needed help to be connected and disconnected to the sleep apnoea device at night—when she had to use the bathroom for example—so home care was not an option. As a result, Annie had to give up living independently. She made a deliberate decision, based on recommendations from her friends, and moved with her dog to a care facility for the elderly situated a short distance from her children. After a short period, the old-fashioned look of the facility and its atmosphere started to irritate her. It looked like a prison to her and there were no shared activities for the residents. The lack of a communal dining room proved to be a big problem for Annie. She needed company because eating with others was important to her. She already lost too much weight due to difficulties eating regularly on her own. Annie refused to unpack her belongings and started looking for another place to live, thus facing another decision-making process. Her first decision was mainly based on information from friends, but this time she first visited potential care facilities and relied on her gut feeling. As she looked around a possible new environment, she was asking herself what sort of care exactly she was looking for and the social atmosphere she wanted to live in. Even living further away from her children was acceptable because most important for her was a ‘click’ with the care facility, being in a social atmosphere with easy access to contact with other residents, and respect for her independence in most activities of daily life. Annie had several preferences, but was not sure which care facility could fulfil her preferences. She was certain that her first decision was the wrong one, and wanted to leave her current care facility as soon as possible.

#### Annie’s case

Annie’s case illustrates an independent woman who is obliged to move to a care facility due to her increasing care needs. The introduction of the apnoea device had changed Annie’s situation. Home care at night was not possible, thus moving to a care facility meant she had to give up her independence. This sudden change made it impossible for Annie to make the right decision about the most appropriate care, and the urgent context led to her following advice from friends. The first decision she made led to a non-preferred situation at the current care facility. Annie had not considered all possibilities while making the decision.

The only influence from calculation was the inadequate home care that had obliged Annie to move. This had an impact on her options. During the decision-making process, Annie did not take much time to acquire knowledge about her options, and its impact on the calculation was low. In her current situation in the care facility, Annie was still not sure about her preferences for an ideal situation. It appeared that the preferences of her friends differed from her own. Annie noticed this almost as soon as she arrived at her current care facility, but she was not sure what she wanted instead. Consequently, she took more time to gain information, by visiting potential care facilities. This information and current experiences helped with the calculation. At first, Annie relied on her gut feeling about care facilities. After experiencing the atmosphere in her current care facility, she activated the calculation. Seemingly different from her intuition, her beloved dog and a good social atmosphere were more important to her than the travelling distance from her children.

If Annie had taken more time to explore her preferences, she might have made a different decision. Assistance with the decision-making process and thereby help in enhancing the calculation that for Annie was the eliciting and prioritization of values, may have been beneficial.

**Box 2. Case description Gilda (mental health care)** Gilda, a 52-year-old highly-gifted woman was searching for a suitable therapy to help her cope with her mental problems and feelings of insecurity. Gilda had two children who were living in another city and did not visit or help her with anything. She asked for a review of her diagnosis of high-giftedness, as this did not make her eligible for specialized care. The resulting diagnoses of giftedness and hypersensitivity did not solve her problem, as the DSM V does not recognize it. However, being labelled as hypersensitive helped Gilda to recognize its effect on her life. The giftedness had already made her life difficult at some points. Gilda longed for contact with people of a similar intellectual level. Conversations with ordinary people often made her anxious or resulted in her becoming depressed. Gilda’s hypersensitivity made her even more insecure which was made worse by her family’s non-acceptance. Gilda tried to manage her hypersensitivity with medication. She only took some prescribed medication whenever she felt it was necessary and the result was unsatisfactory. Gilda had already been prescribed this medication before the diagnosis of hypersensitivity was added to giftedness. As the medications were not working satisfactorily, she needed therapy *“to protect myself against the outside world”*. Her ultimate wish was to have a coach to help her cope with her problems and search for an affordable therapy. Since Gilda could not afford a coach, she searched by herself and tried several options, such as reiki, osho meditation (Bhagwan Sri Rajneesh), mindfulness, and core movement. She was hoping that one of these therapies would help her to manage her hypersensitivity better, so she could have an easier and happier life.

#### Gilda’s case

Gilda’s case illustrates a woman with difficulties finding solutions for her poor ability to cope with giftedness and hypersensitivity. Her diagnoses provided insights into her situation as well as options for therapy. Gilda knew that she wanted to cope with the outside world, but the challenge was to find the appropriate therapy. The context made her willing to manage her hypersensitivity and try therapies. However, Gilda had no intuition about what sort of therapy would be appropriate for her and she was unable to find any therapy on her own. Gilda wanted assistance from professional coaches, but she could not afford them. This meant she did not get any external support, and she could not expect help from family as they stigmatized her and did not accept her as she was.

Guided by her intuition, using information from the internet, and considering recommendations made by acquaintances, Gilda found various therapies and was willing to try them to see if a treatment would fulfil her needs. However, Gilda’s major barriers were insufficient insurance coverage and lack of means. Since the therapies were not covered by insurance and she had little money, it was impossible to try and experience the therapies. Experiences were necessary for self-management and calculation of the construction of preferences.

**Box 3. Case description Esrin (social care)** Esrin, a 28-year-old woman from Kurdistan with learning difficulties, was living with her parents and two brothers. Esrin wanted to live independently and needed help to search for a place to live. Since, due to her learning difficulties, Esrin needed assistance with several situations in her daily life, she was unable to live alone. A formal application was required for admission to a care facility with supervision. The results of a recent IQ test needed to be included in the application form, a test Esrin and her family could not afford. The search had become more urgent because her parents wanted to move to a neighbouring country. Esrin’s brothers claimed that it was her task, as a sister, to take care of the family. She was angry with her father and even more afraid of him, because he threatened to hurt her and her mother. This complex, but difficult to change, situation at home became unbearable for her. After a while and with financial support from a welfare organization, Esrin had an IQ test and proceeded to apply for the formal approval of the care needs she articulated. Her application was reconsidered and she was considered eligible for a care facility where she could live independently under supervision. Together with an independent adviser, she started to search for a facility. As well as place to live independently, Esrin wanted a job as a hairdresser or at an animal shelter, but these expectations were unrealistic. Her learning difficulties were such that she did not have the capacity to hold down such a job, but Esrin did not realize this. Esrin wanted to live in the town where she grew up in the Netherlands. Although Esrin would be happy to leave her parents’ house, it meant she had to leave her mother in an unfavourable situation. The second option was to live close to her family. According to Esrin, this would result in a hopeless, sad situation for her. The third option would be to stay at home with a crisis shelter on hand in case of emergency. This would be unfavourable and emotionally undesirable for Esrin.

#### Esrin’s case

Esrin’s case illustrates she had a complex but stable situation at home, but she desired and expected to live independently and find a job. However, in her situation it was difficult to meet preferences. The situation with her father and brothers was unbearable, but this situation might have changed if her parents had moved when they intended. The prospect of her parents leaving made Esrin think about living on her own. Her calculations were based on experiences and resulted in preferences about places to live.

Although her preferences seemed feasible, they were hard to fulfil due to the lack of formal approval for care. Esrin was unable to include all these aspects in a complete construction of preferences to make a decision. She had preferred and non-preferred options about a suitable place to live. Although these preferences were possibly formed by calculation (experiences from her youth), due to her learning difficulties, her preferences were unrealistic. She needed help to create realistic expectations concerning her options. Esrin’s decision-making could be facilitated by assistance in the phrasing and understanding of realistic preferences, for example options for housing and work.

**Box 4. Case description Lucas (care of people with disabilities)** Lucas is a 54-year-old man with Down syndrome and a developmental age of six. Lucas’ sister was looking for a care facility for Lucas since he still lived with his 86-year-old mother. Although there were several potential care facilities, Lucas preferred to live in one specific facility, because his favourite radio DJ lived nearby. Lucas’s emotional expressions and behaviour underpinned his preference. His sister started the necessary arrangements for Lucas to move to his favoured facility, but Lucas was not taking the final step of moving due to the loyalty he felt towards his mother. His mother told him that she ‘would die’ if he were to move. Lucas was increasingly taking care of his mother instead of the other way around, as she was aged 86 and gradually becoming frail. Lucas’ mother acknowledged the importance of specialized care for Lucas, but wanted him to live near to her home so she could keep in close touch with him. When the preferred apartment for Lucas at his favourite care facility was offered to him he was required to accept the offer within a limited timespan otherwise the apartment would be offered to someone else. According to his sister, moving would be the best for Lucas. She assumed that the idea that Lucas would still be at home when his mother died was unacceptable, because Lucas would not be capable of coping with such a situation. If the apartment in his favoured facility was not available at that time, this would become an emotional challenge, and Lucas could end up in a non-preferred and unfamiliar environment.

#### Lucas’ case

Lucas’ case shows that it is not always the client who directly expresses the preferences. Lucas was not able to explain preferences and make the decision on his own, therefore his family who know and understand him assisted in the decision-making process. Lucas’ context had been stable over recent years while he was still living with his mother. This context was the preferred situation for the mother and partially so for Lucas. According to his sister, Lucas’ preferences were to have a happy mother and to live nearby his favourite DJ. This was her intuition, based on Lucas’ emotional expressions and behaviour.

It was his sister in particular who faced the challenges of the situation and the decision-making, because she thought that moving Lucas to a preferred care facility would be better for him. Lucas’s sister tried to convince her mother, but the mother wanted to have Lucas close by. The challenge was to get Lucas moving. Although his sister’s preference was that he should move, Lucas’ own preference seemed to be not to upset his mother and therefore not move. All family members involved made their own different calculations, the main goal was to achieve consensus.

The situation of Lucas and his family illustrates a construction of preferences that differs between family members. The challenges in this case were not caused by difficulties with the construction of preferences. Both Lucas and his sister would have benefited from assistance to cope with the situation and to reach consensus about options to facilitate the decision-making.

Table 1 gives a summary of the cases presented in this paper based on the construction of preferences: context (stable/unstable) and calculation (possible/impossible). Annie’s case shows that a client who followed her intuition in a certain situation can make a less than optimal decision, and that she needed assistance for the calculation. Gilda’s case illustrates a client in an unstable context who was influenced by her family and the unaffordable cost of therapies, and who was unable to make calculations due to the lack of experience of therapies. Esrin’s case illustrates a client who formulated preferences in a complex stable context, but whose preferences were unrealistic and who needed assistance to construct preferences realistically. Lucas and his family knew their preferences, but nothing happened due to different interests of family members. They would probably not benefit from assistance with the construction of preferences, but needed assistance with the whole situation.

**Table 1:** Key findings based on context and calculation.

	Stable context	Unstable context
**Calculation possible**	*The client has a stable context and calculation is possible* Example case: Lucas • Lives at home with his mother • Needs specialized care • His preferred care facility nearby his favourite DJ is available • Different interests and preferences of family members	*The client has an unstable context and calculation is possible* Example case: Annie • Living in a non-preferred environment and now searching for a different care facility • Following advice from friends versus visiting other care facilities to obtain knowledge • Changing preferences, desires a more social environment
**Calculation impossible/ difficult**	*The client has a stable context and more knowledge is needed for calculation* Example case: Esrin • Complex stable family situation • Learning difficulties, and no formal approval for care • Has non-realistic preferences about living independently and starting a job • Unknown possibilities for supervised housing or work	*The client has an unstable context and more knowledge is needed for calculation* Example case: Gilda • Changing diagnoses and difficulties to manage hypersensitivity • Searching for the preferred therapy by trial and error • No financial support • Desire for assistance (coach)

## Discussion

This study explored the construction of preferences of clients during the decision-making process concerning long-term care. Four cases of people considering this decision were analyzed in accordance with the construction of preferences. The results showed that clients experience different barriers, for example, the context could be influenced by a high reliance on intuition, or a vulnerable family situation, and the calculation by missing experiences, or lack of assistance. In an unstable context, intuition might dominate decision-making, and lesser capabilities of clients make calculations difficult. This study corroborates the importance of focusing on someone’s construction of preferences influenced by context and calculation, and combining these two characteristics is beneficial in decision-making. Annie’s decision was led by context, making her regret it later on. Gilda’s decision was influenced by regulations and gathering information, but calculation was difficult due to missing experiences. Esrin had preferences based on her life, but needed assistance to construct preferences realistically. Lucas and his family knew their preferences, but no actual steps were taken due to other issues.

Clients do not consult quality of care reports, instead they prefer informal information (experiences of others), or information about waiting lists [10]. This study discloses that the process of searching for preferable care can be chaotic and that clients do actively request assistance. When involved in the decision-making process, clients need to be made aware of personal preferences [15]. In the Netherlands, assistance from professionals called independent care coordinators is accessible to all clients, but most clients are not aware of this [16]. The role of an independent care coordinator is to share knowledge about the route a client could follow during the decision-making process and the options a client might have. In addition, recommending guidance in this search, this paper suggests that help with the construction of preference may be beneficial. Esrin needed to balance her preferences according to actual possibilities, and Gilda asked for assistance with her search for a preferred therapy. This assistance in exploring client’s preferences is clearly recognised in the model of shared decision-making [17]. Assisting clients with preferences elicitation could enhance a client priority-based focus trough integration of these preferences [18].

The characteristics of context and calculation are used to gain insight into the consequences of the facilitators and barriers faced by clients during the decision-making process. These two characteristics are comparable to the two systems described by Kahneman [7]. System I–the fast system–is someone’s intuition induced by a situation. Although comparable to context, it takes a narrower view on someone’s situation. System II–the slower system–is not the first reaction of the brain, and needs time for the activation and incorporation of information, which is similar to calculation. This illustrates that in some cases, such as that of Gilda who did not get reimbursement for therapies, that regulations predefine the available options [19].

Although the Dutch health care authorities expect responsible and self-managing clients who articulate preferences, it seems that not all clients fulfil these expectations because it is too difficult for them. Not just authorities, also caregivers are often unaware of the difficulties clients have with decision-making due to limited intellectual abilities [20]. Advocates to represent the best interest of clients with lower intellectual abilities seem useful [21]. Although intellectual abilities influence the decision-making capabilities of a client, this study highlights that the authorities should moderate their expectations, because our study suggest that many clients need assistance with the decision-making process [22]. As shown in Annie’s case, her quality of life decreased due to the decision she made, but with assistance with the subsequent decision, she was able to reflect on her decision and formulate preferences. Some clients request tools to improve their self-management [23]. A tool can increase the awareness that they might need care in the future, and help them to question the important aspects of their life before making the decision [24]. The analysis of the client records in particular showed a number of attempts by care organizations to get a grip on the desirable situation for a client.

This study suggests that clients might need assistance to elucidate preferences. However, this could also be useful for the caregiver to help them understand the preferences of their clients. One possible solution might be a tool to question and phrase someone’s preferences, tailor made to their unique situation. This could assist with eliciting values and formulating important aspects and preferences during the decision-making process. Furthermore, clients could use the tool to explain their personal preferences to caregivers, this could be part of the exploratory discussions the client has with a care organization in order to get a broader view of the aspects clients consider to be important in their lives. Although a tool could assist dialogue, it might not have additional value in cases comparable to that of Lucas, where they know exactly which care organization they prefer.

A methodological strength of this study is the large data set of 95 cases (16 interviews and 79 client records), and the inclusion of actual situations from four care sectors, albeit non-acute situations. The findings might be interpreted as supporting the need for advanced care planning. It was not possible to observe all steps taken by clients during the decision-making process, which is a limitation. Due to the convenience sampling, there was an unequal spread of included participants over the four care sectors, which could have an implication for the validity and generalisability of the findings. Another limitation is the inclusion of four out of twelve provinces in the Netherlands and the different research teams spread over these provinces. Fewer researchers might increase the research efficiency and lower the risk of diversity in data collection.

## Conclusion

This study shows the differing characteristics of clients in need of long-term care, the challenges they face during the decision-making process, and the variable influence of context and calculation. It is useful to discuss whether someone needs assistance with the construction of preferences and how to go about it. Client’s capacity to engage in the decision-making process varies over time, might differ among cases, and is influenced by their circumstances. This will result in a varying need for assistance, a well-designed tool to support the elicitation of preferences would be beneficial to facilitate in the decision-making process.

## Supporting information

S1 File The interview guide in Dutch and English. This is the interview guide used in this study in the original language and in English.
(DOCX)
