# Supplementary material for: Getting to grips with the process of decision-making in long-term care. Descriptive cases illustrate the chaotic reality of the construction of preferences
Source: PLoS One. 2019 May 24;14(5):e0217338. doi: 10.1371/journal.pone.0217338 (PMC6534314; doi:10.1371/journal.pone.0217338)
Supplement: S1 File — This is the interview guide used in this study in the original language and in English. (DOCX) [file pone.0217338.s001.docx]

**S1 File. The interview guide in Dutch and English**

**Kiezen in de langdurige zorg (original in Dutch)**

Algemene kennismaking

1. Bedanken dat cliënt heeft toegezegd deel te willen nemen.
2. Uitleggen doel van dit gesprek:
   1. Kennismaken: wie is wie,
   2. Aanleiding voor zoeken van hulp en hoe het keuzeproces tot nu toe is gegaan;
   3. En ik wil graag weten wat uw indruk is van enkele websites met keuze-informatie.
3. Onderzoeker begint met zichzelf verder voor te stellen:
   1. Naam;
   2. Functie;
   3. Achtergrond wat betreft studie en werkervaring;
   4. Rol binnen het onderzoek;
   5. Vragen of cliënt nog vragen heeft over de onderzoeker.
4. Cliënt stelt zich voor (naam; rest volgt bij vraag …).

Uitleg over het onderzoek

Mensen die gebruik (gaan) maken van langdurige zorg, zoals in een verpleeghuis of in de gehandicaptenzorg, maken keuzes voor welke zorg ze nodig hebben en welke organisatie deze zorg gaat leveren. Het maken van deze keuzes kan ingewikkeld zijn. Wij willen graag weten hoe dit proces precies verloopt bij mensen die daadwerkelijk voor deze keuze staan.

1. Als het goed is, heeft u een informatiebrief ontvangen over het onderzoek. Heeft u nog vragen over het onderzoek naar aanleiding van de informatiebrief?
2. Zo niet, zou u dan dit informed consent formulier tweemaal willen ondertekenen? Eén formulier kunt u zelf houden en één heb ik nodig voor onze administratie.

**Deel A: Vragen over aanleiding voor zoeken van hulp**

De volgende vragen gaan over waarom u zorg aanvraagt en wat daarbij voor u belangrijk is.

1. Graag neem ik vanaf nu het gesprek op. Vindt u dat goed?

*[Indien ja, opname starten en beginnen met inspreken: “Dit interview graag als volgt opslaan: Eerste interview, ID-nummer …”; Indien nee, ijverig meeschrijven.]*

1. Voor welk probleem vraagt u nu zorg aan?
2. Wat is de reden dat u nu zorg aanvraagt?
3. Hoe urgent is de vraag?
4. Welke zorg vraagt u aan?

*[Probeer te achterhalen om welke zorgvorm het gaat, bijv. zelfstandig wonen, verblijf in een instelling, etc.]*

1. Welke opties heeft u al overwogen?

*[Probeer te achterhalen tussen welke zorgvormen de cliënt twijfelt of welke zorgorganisaties al in beeld zijn.]*

1. Hoe zijn deze opties onder uw aandacht gekomen?
2. Met wie heeft u er al over gepraat?
3. Hoe bent u bij deze persoon terecht gekomen?
4. Waar ging het gesprek over?
5. Wat wilde u in dit gesprek te weten komen?
6. Waarop hebt u gelet tijdens het gesprek?
7. Wat bent u in dit gesprek te weten gekomen?
8. In welk stadium van het keuzeproces zit u nu?

*[Bijv. eerste indicatiestellingsgesprek gehad, oriëntatie op mogelijke zorgorganisaties, gesprekken gehad met mogelijke zorgorganisaties, laatste gesprek met beoogde zorgorganisatie gehad, definitieve keuze gemaakt.]*

1. Over hoeveel tijd moet de keuze zijn gemaakt?
2. Waarmee bent u op dit moment (het meest) mee bezig om uw keuze te kunnen maken?

*[Ga door middel van ‘probing’ en ‘prompting’ dieper in op wat de cliënt heeft gezegd om te achterhalen wat de cliënt op dit moment belangrijk vindt bij het maken van een keuze.]*

1. Kunt u me daarover iets meer vertellen?
2. Wat bedoelt u met *[vul een door de cliënt gebruikte term in]*?
3. Wat betekent *[vul een door de cliënt gebruikte term in]* voor u?
4. Kunt u me een voorbeeld geven hoe u dat heeft gedaan?
5. Hoe kwam u erbij om dat te doen?
6. Wat dacht u toen?
7. Welke invloed heeft dit op u gehad?
8. Wat heeft dit u opgeleverd bij het maken van uw keuze?

**Deel B: Vragen over het keuzeproces**

1. Welke vragen heeft u m.b.t. het keuzeproces?;
2. Welke informatie heeft u gezocht of gekregen?
3. Welke afwegingen heeft u gemaakt?
4. Welke inzichten heeft u verkregen?

*[Daarbij telkens verder vragen naar met wie de cliënt het daarover heeft gehad en wat het heeft opgeleverd.]*

**Deel C: Vragen over gebruik van keuze-informatie**

De volgende vragen gaan over informatie die u heeft gebruikt bij het maken van uw keuze.

1. Heeft u informatie (op)gezocht tijdens de voorafgaande periode? Ja/nee

*[Zo nee: ga verder naar deel D.]*

1. Welk soort informatie hebt u gezocht?

*[Bijv. mondeling door mensen die u kent, website van de instelling, andere websites…]*

1. Wat wilde u met deze informatie precies te weten komen?
2. Hoe heeft u naar deze informatie gezocht?
3. Wat was de reden voor u om deze informatie te gaan zoeken?
4. Hoe heeft deze informatie u ondersteunt bij het kiezen van de beste zorgorganisatie of vorm van zorg?
5. Zou u weer gebruik maken van deze informatie als u (opnieuw) een keuze ging maken voor een zorgorganisatie of vorm van zorg? Waarom wel of niet?
6. Heeft u op grond van deze informatie voor een zorgorganisatie of vorm van zorg gekozen? *[Probeer te achterhalen of de cliënt alle kwaliteitskenmerken even belangrijk vindt zonder te sturen met directe vraag.]*

**Deel D: Vragen over websites met keuze informatie**

Graag wil ik met u door een website met keuze-informatie heen lopen, zodat u mij kunt vertellen wat u daarvan vindt.

*[Open de website behorende bij de zorgvorm voor de cliënt.]*

1. Bent u bekend met deze website? Ja/nee

Ik wil u vragen om deze internetpagina te bekijken en te lezen. Zou u tijdens het lezen hardop willen denken? Hardop denken klinkt misschien een beetje vreemd voor u. Het gaat erom dat u hardop meepraat terwijl u denkt. Wij willen gewoon weten wat u vindt en hoe u de informatie begrijpt. Daarna stel ik u enkele vragen over de internetpagina die u gezien heeft. Er zijn geen goede en geen foute antwoorden.

*[Gebruik onderstaande vragen als een ‘open’ interviewleidraad om samen door de website te lopen.]*

1. Wat denkt u?
2. Wat is volgens u de bedoeling van deze website?
3. Zijn er woorden of zinnen die onduidelijk zijn? Ja, welke en waarom?
4. Waaraan denkt u als u het woord “waarderingen” leest?
5. Waaraan denkt u als u naar het cijfer kijkt? Wat betekent dit cijfer voor u?
6. Wie zou deze informatie kunnen helpen?
7. Hoe zou deze informatie kunnen ondersteunen bij het kiezen van de beste zorgorganisatie of vorm van zorg?
8. Zou u gebruik maken van de informatie op deze website als u (opnieuw) een keuze ging maken voor een zorgorganisatie of vorm van zorg? Waarom wel of niet?
9. Zou u op grond van deze informatie de beste zorgorganisatie kiezen en u daar ook laten verzorgen of ondersteunen? Zo ja, welke informatie op deze website zou dan de doorslag geven?

*[Probeer te achterhalen of de cliënt alle kwaliteitskenmerken even belangrijk vindt zonder te sturen met directe vraag.]*

1. Welke informatie zou volgens u op deze website niet mogen ontbreken?
2. Wilt mij nog iets vertellen over deze website wat nog niet aan de orde is geweest en wat u belangrijk vindt?

*[Zet het opnameapparaat uit en ga verder met het invullen van de vragenlijst.]*

Afronden interview

We zijn nu aan het einde van het interview.

Mag ik u nog een keer bellen als ik er achter kom dat ik iets vergeten ben te vragen? *[Noteer of de cliënt hiermee instemt.]*

Heeft u zelf nog vragen of opmerkingen?

*[Bedank de cliënt voor deelname, geef hem/haar een VVV Cadeaubon van €15 en neem afscheid.]*

**Decision-making in long-term care (translated to English)**

General introduction

1. Thank the client for participation.
2. Explanation of the purpose of this conversation:
   1. Becoming acquainted with each other,
   2. Cause to search for care and the decision-making process so far,
   3. Impression of certain websites with decision-making information.
3. Researcher starts introduction him/herself:
   1. Name,
   2. Function,
   3. Background with this study and work experience,
   4. Function within this research,
   5. Ask if client has questions for the researcher.
4. Ask client to introduce him/herself (name, rest will follow with the questions).

Explanation of the research:

People who are in need of long-term care, for example care of elderly and care of people with disabilities, will make decisions considering the care they need and the organization who could provide this care. The process of decision-making might be complicated. The aim of this research is to explore the pathways within this decision-making process of client who face or have faced the decision.

1. You should have received an information letter about this research. Do you have questions about the research after reading this letter?
2. When there are no questions, would you sign this informed consent form twice? You can keep one form for yourself and the other is for our administration.

**Part A: Questions considering the need to search for care.**

De following questions consider the needed care and the things that matter to the client.

1. I would like to start recording this conversation, is this ok?

*[If yes, start recording: “Record this interview: First interview, ID number ….” If no, make extensive notes of the interview.]*

1. For which problem do you need care?
2. What is the cause you require this care from now on?
3. How urgent is the question?
4. Which care are you requesting?

*[Try to track down the care need, for example home care, residential care.]*

1. Which possibilities do you consider?

*[Try to track down if the client is in doubt and whether organizations are already contacted.]*

1. How did you know about these possibilities?
2. With whom did you already talk about this?
3. How did you contacted this person?
4. What did you talk about with him/her?
5. What did you want to know during this conversation?
6. Whereupon did you focus during this conversation?
7. What did you learn during this conversation?
8. In which state of the decision-making are you now?

*[For example, the first indication conversations, orientating about care organizations, conversations with possible organizations, last conversations with preferred organizations, already made the decision.]*

1. When do you need to make the decision?
2. Which things occupy your decision-making the most at this moment?

*[Use probing and prompting to track down the aspects, which matter most during the decision-making.]*

1. Can you tell me a bit more about that?
2. What do you mean with *[use a term used by the participant]*?
3. What does *[use a term used by the participant]* mean to you?
4. Can you give me an example how you did that?
5. How did you come up with that decision?
6. What where your thoughts at that moment?
7. What was the influence on your thoughts?
8. What did it bring for the decision you had to make?

**Part B: Questions considering the decision-making process**

1. What questions do you have considering the decision-making process?
2. Which information did you search or did you receive?
3. Which considerations did you make?
4. Which views did you acquire?

*[Continuously question with whom the participant spoke and the benefit.]*

**Part C: Questions considering the decision-making information**

The following questions consider the information the participant used when making a decision.

1. Did you search for information during the last period?

*[If no, continue with part D.]*

1. Which information did you search?

*[For example from acquaintances, websites of organizations, and other websites.]*

1. What did you want to learn from this information?
2. How did you search for this information?
3. What where the reasons to search for this information?
4. How did this information assist you during the decision-making?
5. Did you choose for a care organization or care need based on this information?

*[Try to track down the importance of quality measures without direct questions.]*

**Part D: Questions considering websites with decision-making information**

I would like to walk through a website with decision-making information, in this way you could tell me you thoughts about this website.

*[Open a website possibly relevant for this participant.]*

1. Are you familiar with this website?

Are you willing to watch and read this website? Would you read and think aloud? Think aloud sounds possibly a bit strange, but it means that you are talking when you are thinking. We would like to know your opinion and understanding of the information. Afterwards I will ask some questions about this website. There are no wrong or right answers.

*[Use the following questions as an open interview guide to walk through the website together.]*

1. What do you think?
2. What is according to you the purpose of this website?
3. Are there sentences or words unclear for you? If yes, which and why?
4. What do you think about when you read the word “appreciation”?
5. What do you think when you see a grade? What does this grade mean to you?
6. For whom would this information be beneficial?
7. How could this information assist when making a decision for a care organization or form?
8. Would you use the information of this website when you had to make a decision (again) for a care organization of care form? Why?
9. Would you be able to make a decision based on the information of this website? If yes, which information has the most decisive power?

*[Try to track down whether the participant considers all quality information even important, without the use of direct questions.]*

1. Which information should not be lacking from the website?
2. Would you like to tell me more about the website that matters to you?

*[Stop the recording device and fill-in the questionnaire.]*

Wrap up the interview

We have reached the end of this interview.

Can I call you once more if I notice I forgot to ask certain questions and miss information? *[If yes, write down contact details.]*

Do you have any questions or remarks?

*[Say thanks to the participant, give the VVV gift-card of €15, and say goodbye.]*
